# Unified field studies of the algae testbed public-private partnership as the benchmark for algae agronomics

**DOI:** 10.1038/sdata.2018.267

**Published:** 2018-11-27

**Authors:** Eric P. Knoshaug, Ed Wolfrum, Lieve M. L. Laurens, Valerie L. Harmon, Thomas A. Dempster, John McGowen

**Affiliations:** 1 National Bioenergy Center, National Renewable Energy Laboratory, Golden, Colorado, 80401, USA; 2 Biosciences Center, National Renewable Energy Laboratory, Golden, CO 80401, USA; 3 Harmon Consulting Inc., 64-5162C Kamamalu Street, Kamuela, HI 96743, USA; 4 Arizona Center for Algae Technology and Innovation, Arizona State University, Mesa, Arizona, 85212, USA

**Keywords:** Agroecology, Biofuels

## Abstract

National scale agronomic projections are an important input for assessing potential benefits of algae cultivation on the future of innovative agriculture. The Algae Testbed Public-Private Partnership was established with the goal of investigating open pond algae cultivation across different geographic, climatic, seasonal, and operational conditions while setting the benchmark for quality data collection, analysis, and dissemination. Identical algae cultivation systems and data analysis methodologies were established at testbed sites across the continental United States and Hawaii. Within this framework, the Unified Field Studies were designed for algae cultivation during all 4 seasons across the testbed network. With increasingly diverse algae research and development, and field deployment strategies, the challenges associated with data collection, quality, and dissemination increase dramatically. The dataset presented here is the complete, curated, climatic, cultivation, harvest, and biomass composition data for each season at each site. These data enable others to do in-depth cultivation, harvest, techno-economic, life cycle, resource, and predictive growth modelling analysis, as well as development of crop protection strategies throughout the algae cultivation industry.

## Background & Summary

Even though algae are at the nexus of the next generation of agricultural innovations for food and fuel production, significant challenges associated with consistent year-round productivity demonstrations at scale remain. Perhaps one of the largest challenges is associated with the lack of a consistent framework for standardized data collection and analysis such that diverse cultivation or pond operational strategies, pond or equipment design, or other associated variables can be compared on a level playing field. Although there are a wide variety of algal cultivation data available in the scientific literature, for example, the Aquatic Species Program closeout report^1^ reviewed algal cultivation research sponsored by the US Department of Energy, none of the literature describes data from the same species grown during the same season at different geographic or climatic conditions or even collected data for a consistent set of metrics. Even though several reports have called for increased research and development of algae technology at scale to address the data gap between assumed and actual experimental values of outdoor pond performance and algal productivity to reduce risk and uncertainty in large-scale deployment, few coordinated projects have been funded^2–5^. Thus the goal of the Algae Testbed Public Private Partnership (ATP^3^) was to provide year-over-year, well-characterized, algal cultivation datasets to the research, development, and commercialization community to help improve the understanding of algae biomass production in terms of geographic and seasonal variation in cultivation, harvest, and productivity to allow for improved techno-economic analysis (TEA), life cycle analysis (LCA), resource assessment (RA), predictive growth modelling, and crop protection strategies for algae cultivation^6^. With the successful establishment of the ATP^3^ algae culturing testbed network, the objective of the Unified Field Studies (UFS) was to address this knowledge gap and generate a robust dataset of algae growth metrics in outdoor open ponds with a focus on the comparison of algae biomass productivity in identical ponds under different seasonal, climatic, and operational conditions at 1000 L scale by controlling the non-geographical related variables of inoculum production, biomass production systems, processes and protocols, system scale, and algae strain.

We report here the datasets from the setup and standardization of the testbed network and the subsequent harvest operations (UFS) of identical algae growth systems across 5 geographic regions over the course of 19 months. This is the first ever demonstration of a multi-site coordinated algae productivity experimental design that required tight management of operational parameters for the cultivation studies, as well as a highly flexible and dynamic environment for data collection that allowed rapid adaptation to user (algae cultivation scientists) feedback. The goal of this paper is to present the complete datasets (Table 1 (available online only)) as well as the cultivation and analytical data collection and curation methods to establish a standardized data collection and curation benchmark for future algae cultivation. By establishing this benchmark, we intend to contribute to the implementation of a set of standardized data metrics for understanding the critical aspects of algae cultivation. A common set of standardized data will facilitate further analysis, discussion, and optimization of algae cultivation as a productive and widespread agricultural crop.

## Methods

We recently published a detailed description of the ATP^3^ framework which covers the experimental and operational alignment for data collection^5,6^. A strong emphasis on the initial validation phase of the project was to ensure that laboratories at different sites were able to carry out the implementation of analytical procedures for data collection in a consistent manner^6,7^. Here, we briefly summarize the essential features of the experiments that produced the data from the UFS, including the location of the sites, the general design of the studies, the equipment used, the algae strains tested, and the typical pond operations during these studies.

### Testbed sites

**Figure 1:**
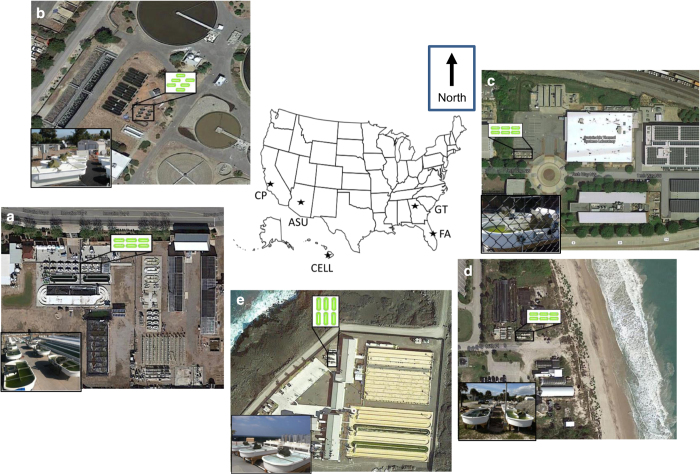
Geographic location, site overview showing pond orientation, installed pond layout (inset diagram), and latitude and longitude of the ATP^3^ testbed facilities. (**a**) ASU: 33.304294, −111.673536, (**b**) CP: 35.254055, −120.674553, (**c**) TRL: 40.824708, −81.862825, (**d**) GT: 33.770844, −84.403457, (**e**) FA: 27.675673, −80.362776, and (**f**) CELL: 19.734646, −156.053119.

Five testbed sites were originally chosen for their differing climatic and geographic locations; Southwest, desert (AzCATI, Arizona State University (ASU), Mesa, AZ); Western, coastal (California Polytechnic State University (CP), San Luis Obispo, CA); Southeast, inland (Georgia Institute of Technology (GT), Atlanta, GA); Pacific, tropical (Cellana LLC (CELL), Kona, HI), and indoor greenhouse (Touchstone Research Labs, TRL); (Fig. 1a–f). Note that TRL was replaced in the ATP^3^ consortium by Southeast, coastal region (Florida Algae (FA), Vero Beach, FL) after the UFS-1.

### Experimental Design

The ponds were installed and a baselining growth experiment without harvesting operations was performed during October to December 2013^6^. With the successful build-out and initial operation of the testbed ponds, the UFS were initiated to determine algae biomass production at each of the geographically diverse sites with three different strains during all four seasons. The experimental design of the UFS was to cultivate algae across the five different geographic regions in the exact same manner. In this way, seasonal and geographic effects could be observed in algae performance data. Pond production experiments were run during each season (identified as approximately 13 week periods) with each individual cultivation experiment lasting at least 8 weeks. The UFS began in April 2014 and ran through July 2015 to conclude this phase of the ATP^3^ project (Table 2). For the purposes of the UFS, we did not attempt to run continuously throughout the entire year to determine the number of pond operational days that could be achieved in a one-year period as this was outside of the scope of the UFS. Rather we intended to explore the seasonal impacts on algae cultivation across the testbed network by running cultivation experiments during each season. Pond crashes caused by fungus (e.g. *Chytridiomycota*), bacteria, other algae (predatory, e.g. *Poterioochromonas*, or competitive, e.g. other eukaryotic algae), and predators (e. g. amoeba) are indicated by underlined values for each experiment (Table 2) and noted in a separate column as a crash in the summary harvest data. The data were collected continuously throughout the experimental duration, periodically checked by each of the site representatives, and ultimately subjected to rigorous quality control (as discussed below in the Data Collection, Review, and Compilation section) when the experiment was completed.

### Algae Strains

The performance of three different algae strains are included in the data reported here: *Nannochloropsis oceanica* KA32 (source: Cellana) grown in a saltwater medium, *Chlorella vulgaris* LRB-AZ-1201 (source: ASU) grown in a freshwater medium, and *Desmodesmus* sp. C046 (source: Cellana), grown in both saltwater and freshwater media. Inoculum for these algae strains was produced in indoor columns using appropriate respective growth media, scaled up, and transferred to outdoor ponds as previously described^6^.

### Pond Design & Operation

At each of the testbed sites, 6 identical raceway ponds measuring 3.5 m by 1.5 m and 35 cm in depth (1025 L nominal volume at 25 cm working depth, with a surface area of 4.2 m^2^ including the paddlewheel area) were installed. Each UFS experiment was performed according to the specific experimental protocol which is included with the dataset as listed in Table 1 (available online only). In general, the experimental plan was designed around a semi-continuous, also known as a drain-and-fill, strategy with harvesting operations to commence once cultures reached a biomass concentration of 0.5 g ash-free dry weight (AFDW) L^−1^. Ponds were operated with 24-hour paddlewheel mixing at 7.2 rpms creating an average flow rate of 9.3 cm s^−1^ with on-demand CO_2_ sparging at 5 L min^−1^ based on pH. Although carbon uptake efficiency is an important metric in terms of algal cultivation, the UFS was setup to ensure CO_2_ was not a limiting reagent and was thus supplied in excess. While the flow rate of CO_2_ to the system was controlled through a pH setpoint, actual volume delivered was not quantified. The initial grow-out period varied but typically took approximately 2 weeks. Harvesting operations to achieve the dilution rate as specified for a given experiment typically involved removing either 25% or 50% of the pond volume three times per week, or 75% of the pond volume once per week. The ponds were harvested using a portable pump and drained to a specified depth depending on the harvesting regime detailed in the protocol for each experiment. Depending on the amount harvested, the time to reset a triplicate set of ponds was typically 45 min including sampling. Since the algal biomass was typically disposed of and not used for downstream processing, harvesting efficiency and percent solids harvested were not calculated and was outside of the scope of the UFS. After routine harvesting, the ponds were re-filled with fresh media to continue the cultivation trial. Ponds were also harvested in response to contamination events and occasionally pre-emptively due to large amounts of rain. Pond samples were taken daily and analysed for water quality and biochemical composition in triplicate according to Table 3.

Compositional analysis methods for total protein, total carbohydrate, and total fatty acid methyl esters (FAMEs) followed consensus methods that were validated and implemented across the ATP^3^ testbed network. These analytical procedures are complex and built on years of experimental analytical biochemistry research and were recently described in detail elsewhere^7,8^. Detailed analysis protocols are available at https://www.nrel.gov/bioenergy/microalgae-analysis.html.

### Data Collection, Review, and Compilation

Researchers at each site were responsible for recording primary experimental data in pre-formatted spreadsheets (available upon request). For any individual experiment (e.g., UFS-1) all spreadsheet formats were the same, but the standard spreadsheet format was modified over time to improve fidelity in recording data. For example, the spreadsheets were set up to automatically highlight outliers (e. g. samples having a relative standard deviation greater than 10%) or identify user input errors (e. g. negative values for pH measurements). The spreadsheets were also occasionally modified to facilitate data entry due to changes in experimental design. For example, the original version of the spreadsheet tracked only the nitrate concentration in ponds while later versions tracked both ammonia and nitrate concentrations (variation in nitrogen source was an integral part of a later experimental design), and the original version of the spreadsheet tracked the optical density of the algae ponds at 750 nm (OD_750_) while later versions tracked both OD_750_ and OD_680_. The final datasets provide columns for nitrate and ammonia and both wavelengths, with “NA” inserted for experiments where these measurements were not taken.

The spreadsheets were shared among ATP^3^ researchers, and resulting datasets underwent multiple rounds of review and correction over the course of the experiment. These reviews identified missing and incorrectly formatted data, and data entry errors. At the conclusion of each experiment, the spreadsheets were transferred to a centralized data repository that was accessible to the core group of data analysts at ATP^3^.

We used the statistical programming language R^9^ (www.R-project.org) to process the data in the spreadsheets into text files. Primary “builder” scripts (one for each data sheet) combined data from the different spreadsheets. Secondary scripts plotted these data to simplify data review and correction. A final script parsed the combined data into separate files for each experiment.

### Code availability

Custom code was written in R to both perform quality control and collate all the data from different spreadsheets. The scripts used to combine, review, and parse the primary spreadsheet data, along with individual primary spreadsheets, are available upon request.

## Data Records

The data from the ATP^3^ UFS are organized into five different types as shown in Table 1 and described in detail below. These data include measurements from routine pond operation, measurements from automated instrumentation, weather data, harvest data, and algae compositional analysis data (Data Citation 1). The instrumentation and weather data are available either as complete data sets or as daily-averaged data.

### Datatype 1: Pond Operational Data

Pond operational data include the measured pond depth (cm), pH, salinity (g L^−1^), pond water temperature (^o^C), nitrogen concentration (mg L^−1^ N), phosphorus concentration (mg L^−1^ P), N:P ratio (molar), the sample ID and tracking ID of a physical sample (if taken), the algae concentration (g L^−1^) as dry weight and ash-free dry weight (AFDW), the ash content of the algal biomass (%), and the OD_750_. The OD_680_ is also available only for UFS-7.

### Datatype 2: Summary Harvest Data

Complete harvest data are available for each experiment at each site. These data include date, strain, batch and source ID, pond treatment, and depth (cm). Harvest data include harvest number, time between harvests, harvest volume (L), amount harvested (g), ash-free dry weight (AFDW; g L^−1^) at the time of harvest, and an indication of a pond crash. The UFS-1 Baseline Experiment was an initial start-up, equipment, and operator trial run thus no harvest data were collected.

### Datatype 3: Compositional Analysis Data

Compositional analysis data were generated on samples taken at regular intervals during each experiment, including all harvest operations. These data include total protein, total carbohydrate, and FAMEs. Individual samples that did not meet the QC requirements or that experienced experimental errors during analysis are not included^7^.

### Datatype 4: Instrumentation Data

The complete and daily-averaged YSI-5200 instrumentation data are available for each experiment at each site. These data include continuous measurement of pH, pond water temperature (^o^C), pond conductivity (ms cm^−1^), dissolved oxygen concentration (DO, mg L^−1^), dissolved oxygen saturation (%), salinity (g L^−1^), and photosynthetically active radiation (PAR, μmol m^−2^ s^−1^). The PAR sensor was connected to only one of the ponds, thus only one pond collected light intensity data, and these data are used to characterize the entire site.

### Datatype 5: Weather Data

The complete and daily-averaged weather data are available for each experiment at each site. These data were collected either on site (ASU and CELL) or using publicly available data in close proximity (within 6 miles) to the site. For the CP site, data from the California Irrigation Management Information System, San Luis Obispo station #52 were used. For the GT site, data from the Clark Atlanta University weather station (http://weather.uga.edu) were used. For the FA site, data from Weather Underground, station: KFLVEROB15 were used. For the TRL site, data from the National Solar Radiation Database delineated by the latitude and longitude of the TRL site were used^10^. These data include air temperature (^o^C), relative humidity (%RH), Global Light Intensity (W m^−2^ s^−1^), daily precipitation (cm), wind speed (km hr^-1^), and wind direction (degrees).

### Datatype 6: Summary Combined Data

The primary Pond Operational Data (*Datatype 1*) and Summary Harvest Data (*Datatype 2*) were combined into one spreadsheet for each experiment to aid in the further analysis of these datasets. Additional metrics beyond primary data collection were also calculated to provide greater utility to the dataset. These calculated metrics include nutrients supplied (g) (nitrate (NO_3_), ammonium (NH_4_), and phosphorous (P)), nutrients utilized (g), nutrient utilization efficiency (%), and nutrient demand (g N or g P g AFDW^−1^). Daily evaporation rate (cm day^−1^) and biomass per unit energy (mg AFDW mol photons^-1^ and mg AFDW kW^−1^) are also included. In addition, pond operator comments are included in these spreadsheets to provide information about what was being observed in the ponds.

## Technical Validation

The experimental protocol document for each UFS provides details regarding procedures on data collection. Routine pond operational measurements (e. g. pH, temperature, optical density) were collected using standard laboratory instruments subjected to regular calibration. Physical samples for AFDW and ash content were collected and analysed in triplicate. The probes used to collect the instrumentation data were calibrated regularly or if a probe was determined to be giving erroneous values. The raw data were plotted using R scripts as noted above as part of the technical validation process. These plots were then manually examined for obvious outliers, for example, a series of pH values rapidly and temporarily changing to values of approximately 4 or 10 indicate pH probe calibration data that were accidentally included in the automated data collection run, or a sudden deviation in temperature when the pond is stable at 30 ^o^C indicating the temperature probe was briefly removed from the pond for cleaning without interrupting data collection. These values were typically flagged with a comment in the primary datasheet. The weather data collected from independently-operated weather stations was not curated by ATP^3^ researchers. Further details of the initial harmonization across the sites and of the technical validation associated with the algae compositional analysis data are presented elsewhere^6,7^.

**Figure 2:**
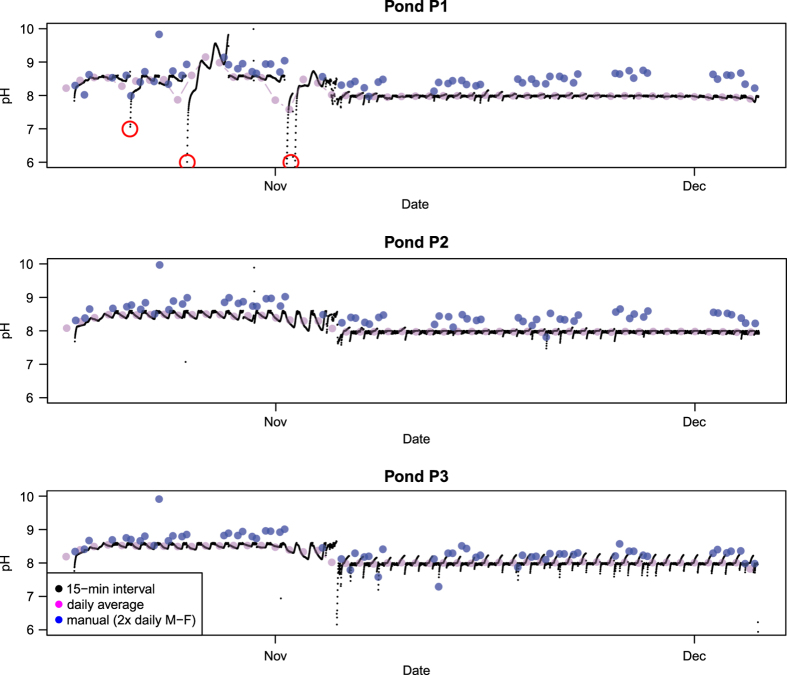
Auto-logged (black), manually-logged (blue), and daily average (magenta) pH in 3 ponds during the UFS-1 experiment at ASU.

We have deliberately not removed large amounts of data, since it is quite difficult to determine with high confidence which data are “good” and which are “bad”. Some of the challenges we experienced in curating these data are illustrated in Fig. 2. The figure shows the pH data collected for three ponds at ASU during the UFS-1 experiment. The black points show the pH measured at 15-minute increments using a pH probe connected to an automated data logger. The magenta points show the daily average value of these pH measurements. The blue points show the manually-recorded pH measurements, collected twice per day (just after sunrise and just before sunset) Monday through Friday along with other pond operational data.

The pH data from the auto-logger (black points) show a small diurnal oscillation due to the change in algae metabolism under illumination and darkness. The daily-averaged data (magenta points) reduce this oscillation. The manually-recorded pH data (blue points) show slightly more variability than the daily-averaged auto-logger data. In the top figure, the auto-logger data show a number of excursions from the apparent “true value” of approximately 8.5. These excursions are highlighted with red boxes in the figure. These excursions in measured pH are due to the inadvertent logging of the pH probe recalibration process, where the probe is removed from the pond and immersed in a series of pH standards. The experimental protocol called for the auto-logger to be paused for the recalibration process. These inadvertent measurements result in slightly lower daily-averaged values as well. The manually-recorded pH data do not show this effect. There appears to be a slight positive bias in the manually-recorded pH data compared to the auto-logger pH data as well, despite being calibrated regularly.

**Figure 3:**
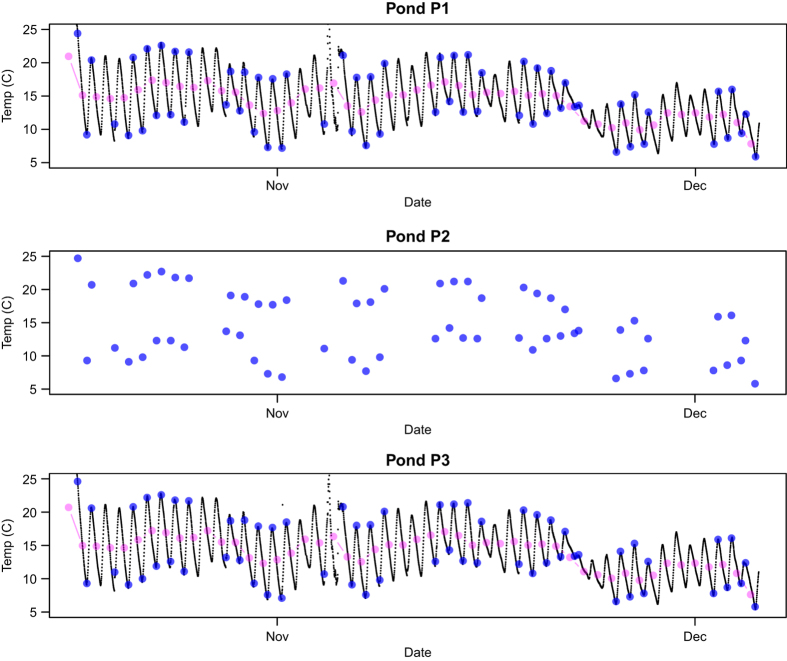
Auto-logged (black), manually-logged (blue), and daily average (magenta) temperature in 3 ponds during the UFS-1 experiment ASU.

In Fig. 3, we show the corresponding pond temperature data, to the pH data in Fig. 2. The black points in Fig. 3 show the temperature measured at 15-minute increments using a temperature probe connected to an automated data-logger. The magenta points show the daily average value of these temperature measurements. The blue points show the manually-recorded temperature measurements, collected twice per day Monday through Friday (morning and evening) along with other pond operational data. The diurnal variation in pond temperature is evident, and the daily-averaged temperature value reduces this variation. The manual temperature measurements are very close to the extreme daily values, since these were made shortly after sunrise and shortly before sunset. Due to a malfunction in the pond P2 auto-logger there are no auto-logger data for pond P2 for this experiment. There is overall a good agreement between the temperature data collected for each of the three ponds, with minimal pond-to-pond variability. There is much better agreement in temperature values as opposed to pH between the manually-recorded data and the auto-logger data.

## Usage Notes

The complete description of a specific UFS experiment requires all five data types as well as the operational protocols (as discussed in the Data Records section). To effectively use these data, it is necessary to link the data from each file. Pond operational data, instrumentation data, and summary harvest data are linked through the site location, pond number, and date and time. Because the weather data and the PAR data characterize the entire site, they can be linked to the pond operational and harvest data through the site location and date and time. Pond operational data and harvest data are linked to the algae compositional data by the use of a tracking ID recorded on the pond operations spreadsheet at the time of harvest, since each biomass sample with compositional analysis data has a unique tracking ID.

Though the datasets were curated for outliers, typographic errors upon data entry, and instrumentation sensor data that was clearly due to a bad sensor, we felt that too much curation could jeopardize the notion that these datasets are purely objective (not selective) datasets. Thus, for example, present in the YSI pond temperature data there are occasional rapid spikes to 40 ^o^C. This represents times when during harvest or other pond operations, the temperature probe was removed and set in a cup of water by the pond. Being in such a small volume of water, the temperature invariably spiked upwards on sunny days. Similarly, as described above, there are multiple instances of the pH measurement for a pond spiking to pH10 or pH4 for several minutes. This was caused by the periodic recalibration of the pH probes while the instrumentation was collecting data. While the protocol called for the instrument to be placed in “standby mode” during the recalibration, this did not always occur.

**Figure 4:**
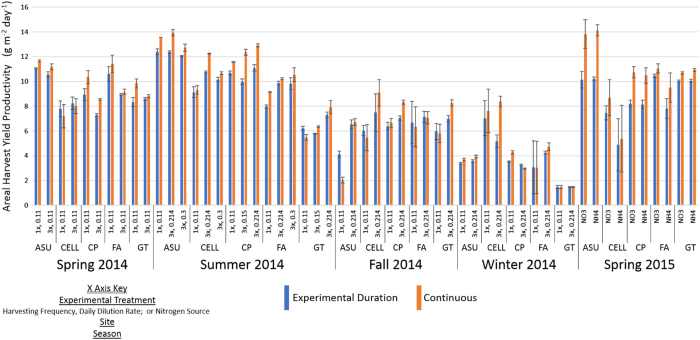
Areal harvest yield productivity for *N. oceanica* KA32 during the entirety of the UFS. Productivity is shown as calculated across the entire experimental duration (blue) and for just the continuous portion of the experiment (orange).

**Figure 5:**
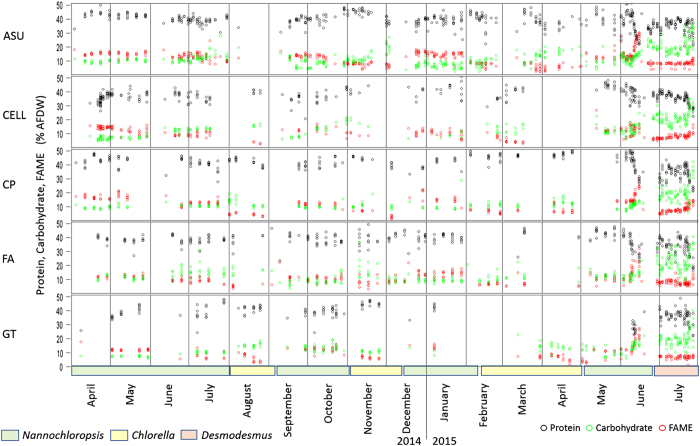
Biomass composition, protein (black), carbohydrate (green), and FAME lipids (red) as % AFDW. The strain of algae is denoted by colored blocks on the x-axis.

It is anticipated that many users of these data will be interested in algae productivity; the rate at which algae biomass is either produced or harvested from outdoor ponds^11^. Algae productivity is generally calculated either as the increase in biomass or the summation of biomass harvested over time. Although a reliable representative measure of growth, biomass productivity calculated as an increase in biomass concentration over time is mainly of academic interest in the context of a biomass production scheme for conversion to biofuels, since it is calculated in such a way that does not account for how much biomass can be or was removed from the pond for conversion to biofuels and may not take into account the subsequent effects of biomass removal. Thus the algae productivity calculation that is more relevant is the calculation based on the actual amount of biomass harvested from the pond and available for conversion to biofuels during the course of a given production run. Usage of the datasets requires careful selection of the appropriate data and additional curation by researchers seeking to use average values for predictive modelling or geographical projections. We have previously presented guidelines and examples on the proper use of the ATP^3^ data to calculate the relevant harvest yield productivity from these datasets^11^ and the harvest yield productivity calculated from these datasets for the algal strain *Nannochloropsis* KA32 is presented (Fig. 4). Clearly the biggest driver of areal harvest yield productivity in *Nannochloropsis* is seasonal with the decrease in temperature and available sunlight with the change from summer into winter. This effect is tempered at the coastal sites of Cellana and Florida Algae. In addition to areal harvest yield productivity, the composition of the algae is a critical parameter in modelling the value and potential of algae cultivation. The algal biomass composition made up of protein, carbohydrate, and lipids as fatty acid methyl esters (FAME) as % AFDW, over the course of the UFS shows how the various components changed through the course of an experiment, across the seasons, and at the various sites (Fig. 5). In general, the UFS was designed to provide baseline growth metrics rather than nutrient deprivation studies. This is evident in the composition as protein is usually high while FAME and carbohydrates are lower. There are examples where nitrogen became depleted with the resulting decrease in protein and increase in FAME and carbohydrates.

Finally, the UFS encompasses typical algal growth at 5 different sites across the United States in regions identified as amenable to outdoor algal cultivation. However, as stated in the introduction, the goal of the Algae Testbed Public Private Partnership (ATP^3^) was to provide year-over-year, well-characterized, algal cultivation datasets. The comprehensive operational, harvest, and composition data, will be useful to the algae research community even though we did not optimize our experiments for either biomass accumulation nor algal compositional shifts, e. g. nitrogen stress to induce the accumulation of lipids. These datasets represent a conservative, non-optimized, estimation of typical algal areal harvest yield productivities and compositions that can be achieved with these strains at the scale, locations, and operational parameters chosen.

## Additional information

**How to cite this article**: Knoshaug, E. P. *et al.* Unified field studies of the algae testbed public-private partnership as the benchmark for algae agronomics Data. 5:180267 doi: 10.1038/sdata.2018.267 (2018).

**Publisher’s note**: Springer Nature remains neutral with regard to jurisdictional claims in published maps and institutional affiliations.

## Supplementary Material



## Figures and Tables

**Table 1:** Listing of complete UFS experimental protocols and data sets.

Experiment Name	Start Date	Web Address	Algal Strain(s)	Experimental Protocol	Datatype 1	Datatype 2	Datatype 3	Datatype 4		Datatype 5		Datatype 6
					Pond Operational Data	Summary Harvest Data	Compositional Analysis Data	Instrumentation Data		Weather Data		Summary Combined Data
								Complete	-Daily-Averaged	Complete	Daily-Averaged	
UFS-1	17-Oct-13	http://en.openei.org/wiki/UFS-1_Results	*N. oceanica* KA32	ATP3_Baseline_UFS_Protocol.pdf	PondOperationalData.OCT172013.KA32.csv	NA	atp3ufs1composition.csv	Instrumentation.OCT172013.KA32.csv	Instrumentation.daily.OCT172013.KA32.csv	Weather.OCT172013.KA32.csv	Weather.daily.OCT172013.KA32.csv	Summary.Combined.OCT172013.KA32.csv
UFS-2	3-Apr-14	http://en.openei.org/wiki/UFS-2_Results	*N. oceanica* KA32	ATP3_Spring_2014_UFS_Protocol.pdf	PondOperationalData.APR032014.KA32.csv	HarvestData.APR032014.KA32.csv	atp3ufs2composition.csv	Instrumentation.APR032014.KA32.csv	Instrumentation.daily.APR032014.KA32.csv	Weather.APR032014.KA32.csv	Weather.daily.APR032014.KA32.csv	Summary.Combined.APR032014.KA32.csv
UFS-3	11-Jun-14	http://en.openei.org/wiki/UFS-3_Results	*N. oceanica* KA32	ATP3_Summer_2014_UFS_Protocol.pdf	PondOperationalData.JUN112014.KA32.csv	HarvestData.JUN112014.KA32.csv	atp3ufs3composition.csv	Instrumentation.JUN112014.KA32.csv	Instrumentation.daily.JUN112014.KA32.csv	Weather.JUN112014.KA32.csv	Weather.daily.JUN112014.KA32.csv	Summary.Combined.JUN112014.KA32.csv
UFS-3	25-Jul-14	http://en.openei.org/wiki/UFS-3_Results	*C. vulgaris* LRB-AZ-1201	ATP3_Summer_2014_UFS_Protocol.pdf	PondOperationalData.JUL252014.LRB-AZ-1201.csv	HarvestData.JUL252014.LRB-AZ-1201.csv	atp3ufs3composition.csv	Instrumentation.JUL252014.LRB-AZ-1201.csv	Instrumentation.daily.JUL252014.LRB-AZ-1201.csv	Weather.JUL252014.LRB-AZ-1201.csv	Weather.daily.JUL252014.LRB-AZ-1201.csv	Summary.Combined.JUL252014.LRB-AZ-1201.csv
UFS-4	10-Sep-14	http://en.openei.org/wiki/UFS-4_Results	*N. oceanica* KA32	ATP3_Fall_2014_UFS_Protocol.pdf	PondOperationalData.SEP102014.KA32.csv	HarvestData.SEP102014.KA32.csv	atp3ufs4composition.csv	Instrumentation.SEP102014.KA32.csv	Instrumentation.daily.SEP102014.KA32.csv	Weather.SEP102014.KA32.csv	Weather.daily.SEP102014.KA32.csv	Summary.Combined.SEP102014.KA32.csv
UFS-4	22-Oct-14	http://en.openei.org/wiki/UFS-4_Results	*C. vulgaris* LRB-AZ-1201	ATP3_Fall_2014_UFS_Protocol.pdf	PondOperationalData.OCT222014.LRB-AZ-1201.csv	HarvestData.OCT222014.LRB-AZ-1201.csv	atp3ufs4composition.csv	Instrumentation.OCT222014.LRB-AZ-1201.csv	Instrumentation.daily.OCT222014.LRB-AZ-1201.csv	Weather.OCT222014.LRB-AZ-1201.csv	Weather.daily.OCT222014.LRB-AZ-1201.csv	Summary.Combined.OCT222014.LRB-AZ-1201.csv
UFS-5	16-Dec-14	http://en.openei.org/wiki/UFS-5_Results	*N. oceanica* KA32	ATP3_Winter_2014_UFS_Protocol.pdf	PondOperationalData.DEC162014.KA32.csv	HarvestData.DEC162014.KA32.csv	atp3ufs5composition.csv	Instrumentation.DEC162014.KA32.csv	Instrumentation.daily.DEC162014.KA32.csv	Weather.DEC162014.KA32.csv	Weather.daily.DEC162014.KA32.csv	Summary.Combined.DEC162014.KA32.csv
UFS-5	29-Jan-15	http://en.openei.org/wiki/UFS-5_Results	*C. vulgaris* LRB-AZ-1201	ATP3_Winter_2014_UFS_Protocol.pdf	PondOperationalData.JAN292015.LRB-AZ-1201.csv	HarvestData.JAN292015.LRB-AZ-1201.csv	atp3ufs5composition.csv	Instrumentation.JAN292015.LRB-AZ-1201.csv	Instrumentation.daily.JAN292015.LRB-AZ-1201.csv	Weather.JAN292015.LRB-AZ-1201.csv	Weather.daily.JAN292015.LRB-AZ-1201.csv	Summary.Combined.JAN292015.LRB-AZ-1201.csv
UFS-6	12-Mar-15	http://en.openei.org/wiki/UFS-6_Results	*C. vulgaris* LRB-AZ-1201	ATP3_Spring_2015_UFS_Protocol.pdf	PondOperationalData.MAR122015.LRB-AZ-1201.csv	HarvestData.MAR122015.LRB-AZ-1201.csv	atp3ufs6composition.csv	Instrumentation.MAR122015.LRB-AZ-1201.csv	Instrumentation.daily.MAR122015.LRB-AZ-1201.csv	Weather.MAR122015.LRB-AZ-1201.csv	Weather.daily.MAR122015.LRB-AZ-1201.csv	Summary.Combined.MAR122015.LRB-AZ-1201.csv
UFS-6	5-May-15	http://en.openei.org/wiki/UFS-6_Results	*N. oceanica* KA32	ATP3_Spring_2015_UFS_Protocol.pdf	PondOperationalData.MAY052015.KA32.csv	HarvestData.MAY052015.KA32.csv	atp3ufs6composition.csv	Instrumentation.MAY052015.KA32.csv	Instrumentation.daily.MAY052015.KA32.csv	Weather.MAY052015.KA32.csv	Weather.daily.MAY052015.KA32.csv	Summary.Combined.MAY052015.KA32.csv
UFS-7	17-Jun-15	http://en.openei.org/wiki/UFS-7_Results	*Desmodesmus* sp. C046	ATP3_Summer_2015_UFS_Protocol.pdf	PondOperationalData.JUN172015.C046.csv	HarvestData.JUN172015.C046.csv	atp3ufs7composition.csv	Instrumentation.JUN172015.C046.csv	Instrumentation.daily.JUN172015.C046.csv	Weather.JUN172015.C046.csv	Weather.daily.JUN172015.C046.csv	Summary.Combined.JUN172015.C046.csv

**Table 2:** Number of UFS pond operational days per strain, site, year, and season.

strain	site	2013	2014	Summer	Fall	Winter	2015		total for UFS
		Fall	Spring				Spring	Summer	
*N. oceanica* KA32	ASU	48.7	55.3	39.8	40.3	42.7	40.7		218.8
	CP	48.7	53.8	39.7	40.8	37.7	39.9		211.9
	CELL	25.7	43.7	35.7	35.7	40.9	32.9		189.0
	FA		48.8	36.7	47.7	41.7	37.8		212.6
	GT	54.8	51.7	34.0	37.7	18.7	34.7		176.8
	TRL	47.7							
*C. vulgaris* LRB-AZ-1201	ASU			25.8	41.7	25.6	49.7		142.9
	CP			34.9	37.7	29.8	55.9		158.3
	CELL			26.6	28.7	27.9	---		83.2
	FA			45.7	47.6	29.7	46.7		169.6
	GT			17.0	26.7	32.0*	52.8		128.4
*Desmodesmus* sp. C046	ASU							39.9	39.9
	CP							39	39.0
	CELL							37.9	37.9
	FA							39.8	39.8
	GT							37.9	37.9
Cells with underline numbers denote experimental runs in which pond crashes occurred.									
*Poor weather conditions continued from the Fall 2014 experimental run thus no Winter experimental run was attempted though weather data was collected for the duration of the experiment.									

**Table 3:** Sample data, units, schedule, and method for data collection.

Sample	Units	Schedule	Sampling Method
OD_750_		Sunrise (+30 min) M-F	Manual
DW, AFDW	g L^−1^	Sunrise (+30 min) M, W, F	Manual
Composition (ash, lipid, carbohydrate, protein)	% AFDW	Sunrise (+30 min) weekly or as change in conditions warrants	Manual
Nutrients (nitrate, phosphate)	mg L^−1^	Sunrise (+30 min) M, W, F	Manual
Weather data (air temperature, % relative humidity, global light energy, precipitation, wind speed and direction)	°C, % RH, W m^−2^, cm, km hr^−1^, degrees	Hourly	Internet weather sites or on-site stations
In-situ sensors (pH, pond water temperature, salinity, % oxygen saturation, PAR)	°C, g L^−1^, %, umol photons m^−2^ s^−1^	15 minute sampling intervals	YSI 5200
Manual pond checks (pH, pond water temperature, depth with paddlewheel off)	^o^C, cm	M - F; AM and PM	Manual
Microscopic check for contaminating organisms	Brightfield, 40× , 100x magnification	Weekly and at final harvest	Manual
Dry weight (DW), photosynthetically active radiation (PAR).			
